# Supplementary material for: High frequency DNA rearrangement at qγ27 creates a novel allele for Quality Protein Maize breeding
Source: Commun Biol. 2019 Dec 10;2:460. doi: 10.1038/s42003-019-0711-0 (PMC6904753; doi:10.1038/s42003-019-0711-0)
Supplement: Supplementary file 1 — Description of Additional Supplementary Files [file 42003_2019_711_MOESM1_ESM.docx]

**Supplementary Data file contains Supplementary Data 1, 2, 3 and 4.**

Supplementary Data 1: Data related to Fig. 3a; average relative level from Bio-Rad CFX-96 thermocycler.

Supplementary Data 2: Data related to Fig. 4d; raw data for the distance measurement of two neighbouring PBs (μm) in *Ra*, *Sab* and *Rabb* endosperm cells by using ImageJ software. The raw data were divided by the conversion factor to get the absolute value in Fig. 4d.

Supplementary Data 3: Data related to Fig. 6e and 6f; raw data for accounting the number of PBs in *αRNAi* and *αRNAi*-M endosperm cells; raw data for the diameter measurement of PBs (μm）in *αRNAi* and *αRNAi*-M endosperm cells by using ImageJ software.

Supplementary Data 4: Data related to Supplementary Fig. 2; raw data for soluble amino acid contents of Lys, Cys and Met in *Rabb*, *αRNAi* and *αRNAi-M* analyzedby the Beijing Mass Spectrometry Medical Research Co. Ltd.
