# Supplementary material for: High frequency DNA rearrangement at qγ27 creates a novel allele for Quality Protein Maize breeding
Source: Commun Biol. 2019 Dec 10;2:460. doi: 10.1038/s42003-019-0711-0 (PMC6904753; doi:10.1038/s42003-019-0711-0)
Supplement: Supplementary file 3 — Supplementary Information [file 42003_2019_711_MOESM3_ESM.pdf]

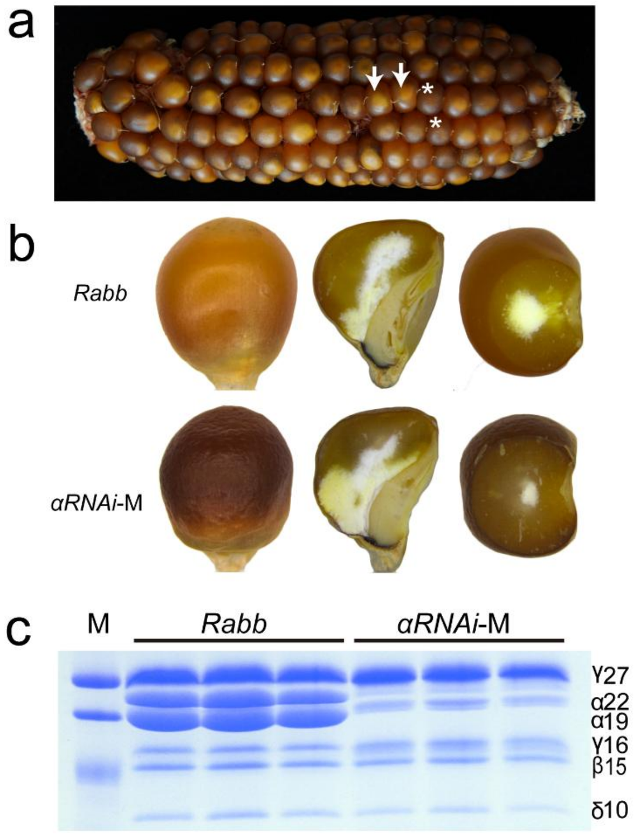

Supplementary Fig. 1 Kernel phenotype and the accumulation of zein proteins in *αRNAi*-M. **a** A self-pollinated *Rabb*/*Rabb*;*αRNAi*/+ ear segregating three quarters of modified kernels (two *Rabb*/*Rabb*;*αRNAi*/+ and one *Rabb*/*Rabb*;*αRNAi*/*αRNAi*) and one quarter of normal kernels (*Rabb*/*Rabb*). Arrows and asterisks indicate normal (*Rabb*) and modified (*αRNAi*-M) kernels; **b** Longitudinal and latitudinal sections of *Rabb* and *αRNAi*-M kernels from the segregating ear in (**a**); **c** SDS-PAGE analysis of zein proteins in *Rabb* and *αRNAi*-M kernels from (**a**). Three kernels for each were analyzed. Each subgroup of zeins is indicated beside the gel. Total zein from 200 μg of corn flour was loaded in each lane. M, protein markers from top to bottom correspond to 25, 20 and 15 kDa.

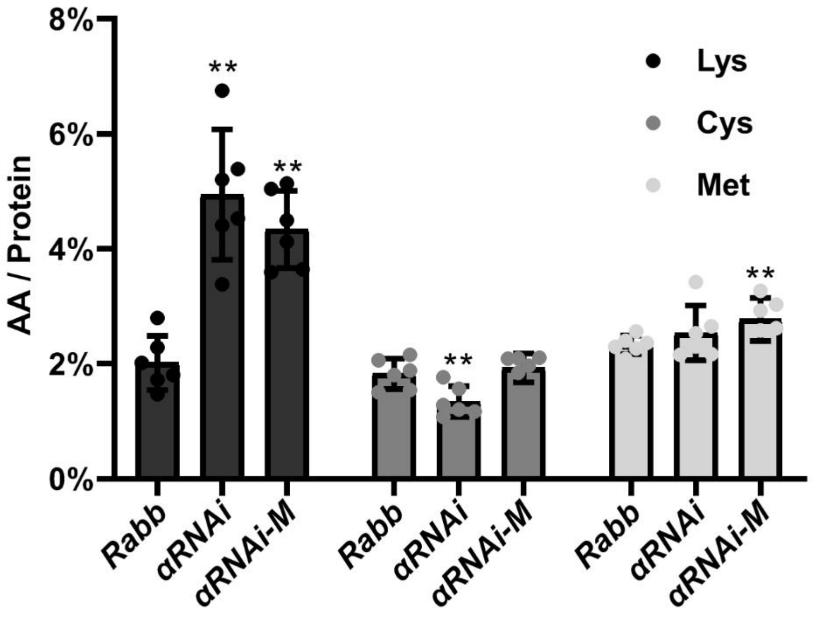

Supplementary Fig. 2 Contents of Lys, Cys and Met in *Rabb*, *αRNAi* and *αRNAi*-M. Error bars show SD from six biological replicates. **P < 0.01 as determined by Student's *t* tests.

Supplementary Table 1. Primer list

Primer Name	Sequences (5′ to 3′)	Note
0707-1F	TAAAGGCCAGCCATATTCTAAA	Insertion primer
0707-1R	GGACTAGCTGAATTTCCTGATG	Insertion primer
γ27-RTF1	TGCCTACAGCCGTCTCG	Quantitative RT-PCR
γ27-RTR1	GAGGGCAACGAGCAACAC	Quantitative RT-PCR
Pbf-F1	GTTAGTGTGCCAGACCGTG	Reference primer for copy number
Pbf-R1	GCTTACTGCAAATGAACTCTC	Reference primer for copy number
0707-2F	TAAAGGCCAGCCATATTCTAAA	Improvement of the 0707-1 primers
0707-2R1	GGACTAGCTGAATTTCCTGATG	Improvement of the 0707-1 primers
0707-2R3	GACCAATGGACACGCTTACC	Improvement of the 0707-1 primers
Solo-F	AGATGTCCAGTGTCGATGGTA	Solo-LTR detection
Solo-R	ATCCATACAACGAGCAATCAA	Solo-LTR detection
KD-F1	AGCCGAGCGATTCAACTAAA	Reference primer for K-D F1
DD-R1	CAAAGGATTCCCGTTCTCAA	Reference primer for K-D R1
KD-F2	TCGTGTTGGGCATTTAAACA	Reference primer for K-D F2
KD-R2	CTTTGACCGGCTAATGGTGT	Reference primer for K-D R2
KD-F3	AAGGGTGGACCCAAATAACC	Reference primer for K-D F3
KD-R3	AAATCTCTAACGGGGCGTTT	Reference primer for K-D R3
KD-F4	GGAGTGAGACAAAGAGTGTCATGT	Reference primer for K-D F4
KD-R4	TCAGGCCACGGTAGTGTTTC	Reference primer for K-D R4
SNP1/1*-F	CGGCAATCAACCTCATGCTG	Rearrangement analysis
SNP1/1*-R	TCCGCTCTTTAGGCTATGCG	Rearrangement analysis
SNP2/2*-F	CGATCGTCCCGCGTCAATA	Rearrangement analysis
SNP2/2*-R	TGTACTTCTGCGTGGCTCAG	Rearrangement analysis
SNP3/3*-F	CACTACCCTACTCAACCGCC	Rearrangement analysis
SNP3/3*-R	TTTCTTCAGTGGGGGACACC	Rearrangement analysis
SNP4/4*-F	GGGTTGTGGATGAGGCGTTA	Rearrangement analysis
SNP4/4*-R	ACTCATCCCACTCTTCTTTTACTCT	Rearrangement analysis
SNP5/5*-F	TGCGGTATGGTTACGCATCT	Rearrangement analysis
SNP5/5*-R	GGTTGCCTATCCAAGTGTGC	Rearrangement analysis
SNP6/6*-F	GGCTTTCCGGACGCTAAATC	Rearrangement analysis
SNP6/6*-R	AGCAGCGATGGTGATATCCT	Rearrangement analysis
SNP7/7*-F	AGATCCAAACAGGGCCTAACA	Rearrangement analysis
SNP7/7*-F	AGCGCTGAATCCAGTTCTGA	Rearrangement analysis
SNP8/8*-F	TGAGTTGATGTGTGCCTGCT	Rearrangement analysis
SNP8/8*-R	TACACAGGCTAGGCTCGAGAT	Rearrangement analysis
