# Supplementary material for: High frequency DNA rearrangement at qγ27 creates a novel allele for Quality Protein Maize breeding
Source: Commun Biol. 2019 Dec 10;2:460. doi: 10.1038/s42003-019-0711-0 (PMC6904753; doi:10.1038/s42003-019-0711-0)
Supplement: Supplementary file 5 — Reporting Summary [file 42003_2019_711_MOESM5_ESM.pdf]

nature research

Corresponding author(s): Yongrui Wu

Last updated by author(s): Nov 21, 2019

Reporting Summary

Nature Research wishes to improve the reproducibility of the work that we publish. This form provides structure for consistency and transparency in reporting. For further information on Nature Research policies, see Authors & Referees and the Editorial Policy Checklist.

Statistics

For all statistical analyses, confirm that the following items are present in the figure legend, table legend, main text, or Methods section.

n/a	Confirmed	
☐	☒	The exact sample size (*n*) for each experimental group/condition, given as a discrete number and unit of measurement
☐	☒	A statement on whether measurements were taken from distinct samples or whether the same sample was measured repeatedly
☐	☒	The statistical test(s) used AND whether they are one- or two-sided<br>*Only common tests should be described solely by name; describe more complex techniques in the Methods section.*
☐	☒	A description of all covariates tested
☐	☒	A description of any assumptions or corrections, such as tests of normality and adjustment for multiple comparisons
☐	☒	A full description of the statistical parameters including central tendency (e.g. means) or other basic estimates (e.g. regression coefficient) AND variation (e.g. standard deviation) or associated estimates of uncertainty (e.g. confidence intervals)
☐	☒	For null hypothesis testing, the test statistic (e.g. *F*, *t*, *r*) with confidence intervals, effect sizes, degrees of freedom and *P* value noted<br>*Give P values as exact values whenever suitable.*
☒	☐	For Bayesian analysis, information on the choice of priors and Markov chain Monte Carlo settings
☐	☒	For hierarchical and complex designs, identification of the appropriate level for tests and full reporting of outcomes
☒	☐	Estimates of effect sizes (e.g. Cohen's *d*, Pearson's *r*), indicating how they were calculated

Our web collection on statistics for biologists contains articles on many of the points above.

Software and code

Policy information about availability of computer code

Data collection	no software was used
Data analysis	Snap Gene(v2.3.2) was used in all sequence analysis; Image J(1.50i) was used in statistics of the number and size of protein body; R (v3.6) was used in all statistical analysis; GraphPad Prism(8.0.2) was used in histogram analysis.

For manuscripts utilizing custom algorithms or software that are central to the research but not yet described in published literature, software must be made available to editors/reviewers. We strongly encourage code deposition in a community repository (e.g. GitHub). See the Nature Research guidelines for submitting code & software for further information.

Data

Policy information about availability of data

All manuscripts must include a data availability statement. This statement should provide the following information, where applicable:

- Accession codes, unique identifiers, or web links for publicly available datasets
- A list of figures that have associated raw data
- A description of any restrictions on data availability

All data used in this study will be made available according to the request.

Field-specific reporting

Please select the one below that is the best fit for your research. If you are not sure, read the appropriate sections before making your selection.

☒ Life sciences ☐ Behavioural & social sciences ☐ Ecological, evolutionary & environmental sciences

Life sciences study design

All studies must disclose on these points even when the disclosure is negative.

Sample size	In order to carry out gene rearrangement frequency statistics, 45,733 individuals were analyzed(sample size, n=45733); To analyze the structural of DNA rearrangement at the γ27 locus, 52 events were sequenced(sample size, n=52); To analyze the number and size of protein bodies(PBs), we performed three biological replicates and statistically and analyzed more than 20 images per sample(sample size, n=360); To quantify γ27 copies in different Uniformmu stocks by genomic real-time PCR, we sequenced three biological replicates for 104 lines (sample size, n=312); For different copy number analysis of γ27, 763 single clone were sequenced(sample size, n=763). For SDS-PAGE analysis, at least 20 kernels from three individual ears were ground into fine flour using steel beads(sample size, n=48); To analyze amino acid content, six biological replicates were performed(sample size, n= 18).
Data exclusions	n/a
Replication	n/a
Randomization	n/a
Blinding	Blinding was not relevant for this study.

Reporting for specific materials, systems and methods

We require information from authors about some types of materials, experimental systems and methods used in many studies. Here, indicate whether each material, system or method listed is relevant to your study. If you are not sure if a list item applies to your research, read the appropriate section before selecting a response.

Materials & experimental systems

n/a	Involved in the study
☒	☐ Antibodies
☒	☐ Eukaryotic cell lines
☒	☐ Palaeontology
☒	☐ Animals and other organisms
☒	☐ Human research participants
☒	☐ Clinical data

Methods

n/a	Involved in the study
☒	☐ ChIP-seq
☒	☐ Flow cytometry
☒	☐ MRI-based neuroimaging
